# Approximation of certain bivariate functions by almost Euler means of double Fourier series

**DOI:** 10.1186/s13660-018-1676-0

**Published:** 2018-04-16

**Authors:** Arti Rathore, Uaday Singh

**Affiliations:** Department of Mathematics, Indian Institute of Technology Roorkee, Roorkee, India

**Keywords:** 40C05, 41A25, 26A15, Double Fourier series, Symmetric partial sums, Modulus of continuity, Modulus of smoothness, Lipschitz class, Zygmund class, Almost Euler means

## Abstract

In this paper, we study the degree of approximation of 2*π*-periodic functions of two variables, defined on $T^{2}=[-\pi,\pi]\times[-\pi,\pi]$ and belonging to certain Lipschitz classes, by means of almost Euler summability of their Fourier series. The degree of approximation obtained in this way depends on the modulus of continuity associated with the functions. We also derive some corollaries from our theorems.

## Introduction

Let $f(x,y)$ be a 2*π*-periodic function in each variable and Lebesgue integrable over the two-dimensional torus $T^{2}=[-\pi,\pi]\times [-\pi,\pi]$. Then the double trigonometric Fourier series of $f(x,y)$ is defined by

1 $$ f(x,y)\sim\sum_{k=-\infty}^{\infty}\sum _{l=-\infty}^{\infty}\hat {f}(k,l)e^{i(kx+ly)}, $$

where

$$ \hat{f}(k,l)=\frac{1}{(2\pi)^{2}} \int^{\pi}_{-\pi} \int^{\pi}_{-\pi }f(u,v)e^{-i(ku+lv)}\,du\,dv $$

are the Fourier coefficients of the function *f*.

The double sequence of symmetric rectangular partial sums associated with Fourier series of *f* is given by

$$ s_{mn}(x,y)=\sum_{k=-m}^{m}\sum _{l=-n}^{n}\hat{f}(k,l)e^{i(kx+ly)}, $$

and its integral representation is given by

2 $$ s_{mn}(x,y)=\frac{1}{\pi^{2}} \int_{-\pi}^{\pi} \int_{-\pi}^{\pi }f(x+u,y+v)D_{m}(u)D_{n}(v) \,du\,dv, $$

where $D_{k}(t)=\frac{\sin(k+\frac{1}{2})t}{2\sin(t/2)}$ is the Dirichlet kernel.

The concept of almost convergence of sequences was introduced and studied by G.G. Lorentz in 1948 [1]. A sequence $\{x_{n}\}$ is said to be almost convergent to a limit *L*, if

$$ \lim_{n\rightarrow\infty}\frac{1}{n+1}\sum_{k=r}^{r+n}x_{k}=L \quad\mbox{for all } r\in \mathbb{N}. $$

Móricz and Rhoades [2] extended the definition of almost convergence to double sequences of real numbers $\{x_{mn}\}$, almost converging to *L*, if

$$ \lim_{m,n\rightarrow\infty}\frac{1}{(m+1)(n+1)}\sum_{k=q}^{q+m} \sum_{l=r}^{r+n}x_{kl}=L \quad\mbox{for all } q,r\in \mathbb{N}. $$

The Euler means $E_{mn}(x,y)$ of the sequence $\{s_{kl}(x,y)\}$ are defined by

$$ E_{mn}(x,y)=\frac{1}{(1+q_{1})^{m}(1+q_{2})^{n}}\sum_{k=0}^{m} \sum_{l=0}^{n}{m\choose k} {n\choose l}{q_{1}}^{m-k}{q_{2}}^{n-l} s_{kl}(x,y),\quad q_{1},q_{2}>0, $$

and almost Euler means of the sequence $\{s_{kl}(x,y)\}$ are defined by

$$ \tau_{mn}^{rs}(x,y)=\frac{1}{(1+q_{1})^{m}(1+q_{2})^{n}}\sum _{k=0}^{m}\sum_{l=0}^{n} {m\choose k}{n\choose l}{q_{1}}^{m-k}{q_{2}}^{n-l} S_{kl}^{rs}(x,y), $$

where

$$ S_{kl}^{rs}(x,y)=\frac{1}{(k+1)(l+1)}\sum _{\gamma=r}^{r+k}\sum_{\mu =s}^{s+l}s_{\gamma\mu}(x,y). $$

The following function classes are well known in the literature (see [3, 4]). For $0<\alpha\leq1$, the Lipschitz class Lip*α* is defined by

$$ \operatorname{Lip} \alpha=\bigl\{ f:T^{2}\rightarrow\mathbb{R} \mid \omega (f,\delta)=O\bigl({\delta}^{\alpha}\bigr)\bigr\} , $$

where $\omega(f,\delta)$ is the modulus of continuity of *f*, defined by

$$ \omega(f,\delta)=\sup_{x,y}\sup_{{(h^{2}+{\eta}^{2})}^{1/2}\leq\delta} \bigl\{ \big|f(x+h,y+\eta)-f(x,y)\big| \bigr\} . $$

For $0<\alpha,\beta\leq1$, the Lipschitz class $\operatorname {Lip}(\alpha,\beta)$ is defined by

$$ \operatorname{Lip}(\alpha,\beta)=\bigl\{ f:T^{2}\rightarrow\mathbb{R} \mid \omega_{1,x}(f,u)=O\bigl(u^{\alpha}\bigr) \mbox{ and } \omega_{1,y}(f,v)=O\bigl(v^{\beta}\bigr)\bigr\} , $$

where $\omega_{1,x}(f,u)$ and $\omega_{1,y}(f,v)$ are the partial moduli of continuity of *f*, defined by

$$ \omega_{1,x}(f,u)=\sup_{x,y}\sup_{|h|\leq u} \bigl\{ \big|f(x+h,y)-f(x,y)\big| \bigr\} $$

and

$$ \omega_{1,y}(f,v)=\sup_{x,y}\sup_{|\eta|\leq v} \bigl\{ \big|f(x,y+\eta )-f(x,y)\big| \bigr\} . $$

For $0< \alpha, \beta\leq2$, the Zygmund class $\operatorname {Zyg}(\alpha,\beta)$ is defined by

$$ \operatorname{Zyg}(\alpha,\beta)=\bigl\{ f:T^{2}\rightarrow\mathbb{R} \mid \omega_{2,x}(f,u)=O\bigl(u^{\alpha}\bigr) \mbox{ and } \omega_{2,y}(f,v)=O\bigl(v^{\beta}\bigr)\bigr\} , $$

where $\omega_{2,x}(f,u)$ and $\omega_{2,y}(f,v)$ are the partial moduli of smoothness of *f*, defined by

$$ \omega_{2,x}(f,u)=\sup_{x,y}\sup_{|h|\leq u} \bigl\{ \big|f(x+h,y)+f(x-h,y)-2f(x,y)\big|\bigr\} $$

and

$$ \omega_{2,y}(f,v)=\sup_{x,y}\sup_{|\eta|\leq v} \bigl\{ \big|f(x,y+\eta)+f(x,y-\eta )-2f(x,y)\big|\bigr\} . $$

Here, we generalize the definitions of $\operatorname{Lip}(\alpha,\beta )$ and $\operatorname{Zyg}(\alpha,\beta)$ given in [3] and [4], respectively, by introducing a new Lipschitz class $\operatorname{Lip}(\alpha,\beta; p)$ and a Zygmund class $\operatorname {Zyg}(\alpha,\beta; p)$.

Let $L^{p}(T^{2})$ ($p\geq1$) denote the spaces of Lebesgue functions on the torus $T^{2}$, with the norm defined by

$$ \bigl\| f \bigr\| _{p}= \left \{ \textstyle\begin{array}{l@{\quad}l} (\frac{1}{2\pi}\int_{0}^{2\pi}\int_{0}^{2\pi} \vert f(x,y) \vert ^{p}\,dx\,dy )^{\frac{1}{p}}, & \hbox{$p\geq1$;} \\ \operatorname{ess} \sup_{0\leq x,y\leq2\pi} \vert f(x,y) \vert , & \hbox{$p=\infty$.} \end{array}\displaystyle \right . $$

Let $f(x,y)$ be a 2*π*-periodic function in each variable belonging to $L^{p}(T^{2})$ ($p\geq1$) class. Then the total integral modulus of continuity of *f* is defined by

$$ \omega_{1}^{p}(f,u,v)=\sup_{|h|\leq u,|\eta|\leq v} \bigl\{ \big\| f(x+h,y+\eta )-f(x,y)\big\| _{p} \bigr\} $$

while the two partial integral moduli of continuity of *f* are defined by

$$ \omega_{1,x}^{p}(f,u)=\omega_{1}^{p}(f,u,0)= \sup_{|h|\leq u} \bigl\{ \big\| f(x+h,y)-f(x,y)\big\| _{p} \bigr\} $$

and

$$ \omega_{1,y}^{p}(f,v)=\omega_{1}^{p}(f,0,v)= \sup_{|\eta|\leq v} \bigl\{ \big\| f(x,y+\eta)-f(x,y)\big\| _{p} \bigr\} . $$

The Lipschitz class $\operatorname{Lip}(\alpha,\beta;p)$ ($p\geq1$) for $\alpha,\beta\in(0,1]$ is defined as

3 $$ \operatorname{Lip}(\alpha,\beta; p):=\bigl\{ f\in L^{p} \bigl(T^{2}\bigr) \mid\omega _{1,x}^{p}(f,u)=O \bigl(u^{\alpha}\bigr) \mbox{ and } \omega_{1,y}^{p}(f,v)=O \bigl(v^{\beta}\bigr)\bigr\} . $$

We also use the notion of integral modulus of smoothness. The total integral modulus of smoothness of a function *f* is defined by

$$ \omega_{2}^{p}(f,u,v)=\sup_{|h|\leq u,|\eta|\leq v} \bigl\{ \big\| \phi _{x,y}(h,\eta)\big\| _{p} \bigr\} , $$

where

$$\begin{aligned} \phi_{x,y}(h,\eta)={}&f(x+h,y+\eta)+f(x-h,y+\eta)+f(x+h,y-\eta) \\ &+f(x-h,y-\eta)-4f(x,y). \end{aligned}$$

The partial integral moduli of smoothness are defined by

$$ \omega_{2,x}^{p}(f,u)=\frac{1}{2}\omega_{2}^{p}(f,u,0)= \sup_{|h| \leq u}\bigl\{ \big\| f(x+h,y)+f(x-h,y)-2f(x,y)\big\| _{p} \bigr\} $$

and

$$ \omega_{2,y}^{p}(f,v)=\frac{1}{2}\omega_{2}^{p}(f,0,v)= \sup_{|\eta|\leq v}\bigl\{ \big\| f(x,y+\eta)+f(x,y-\eta)-2f(x,y) \big\| _{p}\bigr\} . $$

It is clear that $\omega_{2}^{p}(f,u,v)$, $\omega_{2,x}^{p}(f,u)$ and $\omega _{2,y}^{p}(f,v)$ are nondecreasing functions in *u* and *v* and that

$$ 2 \max\bigl\{ \omega_{2,x}^{p}(f,u), \omega_{2,y}^{p}(f,v) \bigr\} \leq\omega _{2}^{p}(f,u,v)\leq2 \bigl\{ \omega_{2,x}^{p}(f,u)+ \omega_{2,y}^{p}(f,v) \bigr\} $$

and

4 $$ \omega_{2,x}^{p}(f,u)\leq2 \omega_{1,x}^{p}(f,u),\qquad \omega_{2,y}^{p}(f,v) \leq 2 \omega_{1,y}^{p}(f,v). $$

For $0< \alpha, \beta\leq2$, the Zygmund class $\operatorname {Zyg}(\alpha,\beta;p)$ ($p\geq1$) is defined as

$$ \operatorname{Zyg}(\alpha,\beta;p):=\bigl\{ f\in L^{p} \bigl(T^{2}\bigr) \mid\omega _{2,x}^{p}(f,u)=O \bigl(u^{\alpha}\bigr) \mbox{ and } \omega_{2,y}^{p}(f,v)=O \bigl(v^{\beta}\bigr)\bigr\} . $$

From (4) it is clear that $\operatorname{Lip}(\alpha,\beta ;p)\subseteq\operatorname{Zyg}(\alpha,\beta;p)$ for $0<\alpha,\beta\leq 1$, and similar to one-dimensional case, $\operatorname{Lip}(\alpha ,\beta;p)= \operatorname{Zyg}(\alpha,\beta;p)$ for $0<\alpha,\beta<1$, but $\operatorname{Lip}(\alpha,\beta;p)\neq\operatorname{Zyg}(\alpha ,\beta;p)$ for $\max(\alpha,\beta)=1$ (see, e.g., [5], p. 44).

Let $\omega(\delta)$ be a nondecreasing function of $\delta\geq0$. Then $\omega(\delta)$ is of the first kind if

5 $$ \int_{\delta}^{\pi}\frac{\omega(u)}{u^{2}}\,du=O \biggl\{ \frac{\omega(\delta )}{\delta} \biggr\} , \quad0< \delta\leq\pi, $$

and $\omega(\delta)$ is of the second kind if

6 $$ \int_{\delta}^{\pi}\frac{\omega(u)}{u^{2}}\,du=O \biggl\{ \frac{\omega(\delta )}{\delta}\log\frac{\pi}{\delta} \biggr\} , \quad0< \delta\leq\pi $$

(see [3]).

A function $f(x,y)$ is said to belong to the class $\operatorname {Lip}(\psi(u,v);p)$ ($p>1$) if

$$ \big|f(x+u,y+v)-f(x,y)\big|\leq M \biggl(\frac{\psi(u,v)}{({u.v})^{1/p}} \biggr), $$

where $\psi(u,v)$ is a positive increasing function of the variables *u*, *v* and *M* is a positive constant independent of *x*, *y*, *u*, and *v* (see [6–8]).

Here, we generalize the definition of $\operatorname{Lip}(\psi(u,v);p)$ ($p>1$) class given above by introducing a new Lipschitz class $\operatorname{Lip}(\psi(u,v))_{L^{p}}$ ($p>1$) defined as

7 $$ \big\| f(x+u,y+v)-f(x,y)\big\| _{p}\leq M \biggl(\frac{\psi(u,v)}{({u.v})^{1/p}} \biggr). $$

Throughout this paper we shall use the following notations:

8 $$\begin{aligned}& \begin{aligned}\phi_{x,y}(u,v)={}&\bigl\{ f(x+u,y+v)+f(x-u,y+v)+f(x+u,y-v) \\ &+f(x-u,y-v)-4f(x,y)\bigr\} ,\end{aligned} \\& S_{k}^{r}(u)=\frac{\sin((k+1)\frac{u}{2})\sin((k+2r+1)\frac{u}{2})}{\sin ^{2}(u/2)}=\sum _{\gamma=r}^{r+k}D_{\gamma}(u), \end{aligned}$$

9 $$\begin{aligned}& S_{l}^{s}(v)=\frac{\sin((l+1)\frac{v}{2})\sin((l+2s+1)\frac{v}{2})}{\sin ^{2}(v/2)}=\sum _{\mu=s}^{s+l}D_{\mu}(v), \end{aligned}$$

10 $$\begin{aligned}& R_{m}^{r}(u)=\sum _{k=0}^{m}{m\choose k} \frac{q_{1}^{m-k}}{(k+1)}S_{k}^{r}(u),\quad q_{1}>0, \end{aligned}$$

11 $$\begin{aligned}& R_{n}^{s}(v)=\sum _{l=0}^{n}{n\choose l} \frac{q_{2}^{n-l}}{(l+1)}S_{l}^{s}(v),\quad q_{2}>0. \end{aligned}$$

### Note 1

We can easily prove that $\phi_{x,y}(u,v)$ satisfies the following inequalities:

12 $$ \big|\phi_{x,y}(u,v)\big|\leq2 \bigl(\omega_{2,x}(f,u)+ \omega_{2,y}(f,v) \bigr) $$

and

13 $$ \big\| \phi_{x,y}(u,v)\big\| _{p}\leq2 \bigl(\omega_{2,x}^{p}(f,u)+ \omega _{2,y}^{p}(f,v) \bigr). $$

Móricz and Xianlianc Shi [4] studied the rate of uniform approximation of a 2*π*-periodic continuous function $f(x,y)$ in the Lipschitz class $\operatorname{Lip}(\alpha,\beta)$ and in the Zygmund class $\operatorname{Zyg}(\alpha,\beta)$, $0<\alpha,\beta\leq1$, by Cesàro means $\sigma_{mn}^{\gamma\delta}$ of positive order of its double Fourier series. They also obtained the result for conjugate function by using the corresponding Cesàro means.

Further, Móricz and Rhoades [9] studied the rate of uniform approximation of $f(x,y)$ in Lip*α*, $0<\alpha \leq1$, class by Nörlund means of its Fourier series. After that, Móricz and Rhoades [10] studied the rate of uniform approximation of a continuous function $f(x,y)$ in the Lipschitz class $\operatorname{Lip}(\alpha,\beta)$ and in the Zygmund class $\operatorname{Zyg}(\alpha,\beta)$, $0<\alpha,\beta\leq1$, by Nörlund means of its Fourier series. In [10], they also obtained the result for a conjugate function by using the corresponding Nörlund means.

Mittal and Rhoades [3] generalized the results of [9, 10], and [4] for a 2*π*-periodic continuous function $f(x,y)$ in the Lipschitz class $\operatorname{Lip}(\alpha,\beta)$ and in the Zygmund class $\operatorname{Zyg}(\alpha,\beta)$, $0<\alpha,\beta \leq1$, by using rectangular double matrix means of its double Fourier series. Lal [11, 12] obtained results for double Fourier series using double matrix means and product matrix means.

Also, Khan [6] obtained the degree of approximation of functions belonging to the class $\operatorname{Lip}(\psi(u,v);p)$ ($p > 1$) by Jackson type operator. Further, Khan and Ram [8] determined the degree of approximation for the functions belonging to the class $\operatorname{Lip}(\psi(u,v);p)$ ($p > 1$) by means of Gauss–Weierstrass integral of the double Fourier series of $f(x,y)$. Khan et al. [7] extended the result of Khan [6] for *n*-dimensional Fourier series. In [13], Krasniqi determined the degree of approximation of the functions belonging to the class $\operatorname{Lip}(\psi(u,v);p)$ ($p>1$) by Euler means of double Fourier series of a function $f(x,y)$. In fact, he generalized the result of Khan [14] for two-dimensional and for *n*-dimensional cases.

## Main results

In this paper, we study the problem in more generalized function classes defined in Sect. 1 and determine the degree of approximation by almost Euler means of the double Fourier series. More precisely, we prove the following theorem.

### Theorem 2.1

*Let* $f(x,y)$ *be a* 2*π*-*periodic function in each variable belonging to* $L^{p}(T^{2})$ ($1\leq p<\infty$). *Then the degree of approximation of* $f(x,y)$ *by almost Euler means of its double Fourier series is given by*:

(i) *If both* $\omega_{2,x}^{p}$ *and* $\omega_{2,y}^{p}$ *are of the first kind*, *then*
  $$ \big\| \tau_{mn}^{rs}(x,y)-f(x,y)\big\| _{p}=O \biggl( \omega_{2,x}^{p} \biggl(f,\frac {1}{m+1} \biggr)+ \omega_{2,y}^{p} \biggl(f,\frac{1}{n+1} \biggr) \biggr). $$
(ii) *If* $\omega_{2,x}^{p}$ *is of the first kind and* $\omega _{2,y}^{p}$ *is of the second kind*, *then*
  $$ \big\| \tau_{mn}^{rs}(x,y)-f(x,y)\big\| _{p}=O \biggl( \omega_{2,x}^{p} \biggl(f,\frac {1}{m+1} \biggr)+\log\bigl( \pi(n+1)\bigr)\omega_{2,y}^{p} \biggl(f,\frac{1}{n+1} \biggr) \biggr). $$
(iii) *If* $\omega_{2,x}^{p}$ *is of the second kind and* $\omega _{2,y}^{p}$ *is of the first kind*, *then*
  $$ \big\| \tau_{mn}^{rs}(x,y)-f(x,y)\big\| _{p}=O \biggl(\log \bigl(\pi(m+1)\bigr)\omega _{2,x}^{p} \biggl(f, \frac{1}{m+1} \biggr)+\omega_{2,y}^{p} \biggl(f, \frac {1}{n+1} \biggr) \biggr). $$
(iv) *If both* $\omega_{2,x}^{p}$ *and* $\omega_{2,y}^{p}$ *are of the second kind*, *then*
  $$\begin{aligned} \big\| \tau_{mn}^{rs}(x,y)-f(x,y)\big\| _{p}={}&O \biggl(\log \bigl(\pi(m+1)\bigr)\omega _{2,x}^{p} \biggl(f, \frac{1}{m+1} \biggr) \\ &+\log\bigl(\pi(n+1)\bigr)\omega_{2,y}^{p} \biggl(f,\frac{1}{n+1} \biggr) \biggr).\end{aligned} $$

For $p=\infty$, the partial integral moduli of smoothness $\omega _{2,x}^{p}$ and $\omega_{2,y}^{p}$ reduce to the moduli of smoothness $\omega_{2,x}$ and $\omega_{2,y}$, respectively. Thus, for $p=\infty$, we have the following theorem.

### Theorem 2.2

*Let* $f(x,y)$ *be a* 2*π*-*periodic function in each variable belonging to* $L^{\infty}(T^{2})$. *Then the degree of approximation of* $f(x,y)$ *by almost Euler means of its double Fourier series is given by*:

(i) *If both* $\omega_{2,x}$ *and* $\omega_{2,y}$ *are of the first kind*, *then*
  $$ \big\| \tau_{mn}^{rs}(x,y)-f(x,y)\big\| _{\infty}=O \biggl( \omega_{2,x} \biggl(f,\frac {1}{m+1} \biggr)+\omega_{2,y} \biggl(f,\frac{1}{n+1} \biggr) \biggr). $$
(ii) *If* $\omega_{2,x}$ *is of the first kind and* $\omega_{2,y}$ *is of the second kind*, *then*
  $$ \big\| \tau_{mn}^{rs}(x,y)-f(x,y)\big\| _{\infty}=O \biggl( \omega_{2,x} \biggl(f,\frac {1}{m+1} \biggr)+\log\bigl(\pi(n+1)\bigr) \omega_{2,y} \biggl(f,\frac{1}{n+1} \biggr) \biggr). $$
(iii) *If* $\omega_{2,x}$ *is of the second kind and* $\omega_{2,y}$ *is of the first kind*, *then*
  $$ \big\| \tau_{mn}^{rs}(x,y)-f(x,y)\big\| _{\infty}=O \biggl(\log \bigl(\pi(m+1)\bigr)\omega _{2,x} \biggl(f,\frac{1}{m+1} \biggr)+ \omega_{2,y} \biggl(f,\frac{1}{n+1} \biggr) \biggr). $$
(iv) *If both* $\omega_{2,x}$ *and* $\omega_{2,y}$ *are of the second kind*, *then*
  $$\begin{aligned} \big\| \tau_{mn}^{rs}(x,y)-f(x,y)\big\| _{\infty}={}&O \biggl(\log \bigl(\pi(m+1)\bigr)\omega _{2,x} \biggl(f,\frac{1}{m+1} \biggr) \\ &+ \log\bigl(\pi(n+1)\bigr)\omega_{2,y} \biggl(f,\frac{1}{n+1} \biggr) \biggr).\end{aligned} $$

### Theorem 2.3

*Let* $f(x,y)$ *be a* 2*π*-*periodic function in each variable belonging to the class* $\operatorname{Lip}(\psi(u,v))_{L^{p}}$ ($p>1$). *If the positive increasing function* $\psi(u,v)$ *satisfies the condition*

14 $$ (uv)^{-\sigma}\psi(u,v) \textit{ is nondecreasing for some } 1/p< \sigma< 1, $$

*then the degree of approximation of* $f(x,y)$ *by almost Euler means of its double Fourier series is given by*

$$\begin{aligned} \big\| \tau_{mn}^{rs}(x,y)-f(x,y)\big\| _{p}={}&O \biggl\{ \bigl((m+1) (n+1) \bigr)^{1/p} \biggl[\psi \biggl(\frac{1}{{m+1}}, \frac{1}{{n+1}} \biggr) \\ &+(n+1)^{-\sigma}\psi \biggl(\frac{1}{m+1},\pi \biggr)+(m+1)^{-\sigma}\psi \biggl(\pi, \frac {1}{n+1} \biggr)\\ &+ \bigl((m+1) (n+1) \bigr)^{-\sigma} \biggr] \biggr\} . \end{aligned}$$

For $p=\infty$, the class $\operatorname{Lip}(\psi(u,v))_{L^{p}}$ reduces to the class $\operatorname{Lip}(\psi(u,v))_{L^{\infty}}$, defined as

$$ \big|f(x+u,y+v)-f(x,y)\big|\leq M\psi(u,v). $$

Thus, for $p=\infty$, we have the following theorem.

### Theorem 2.4

*Let* $f(x,y)$ *be a* 2*π*-*periodic function in each variable belonging to the class* $\operatorname{Lip}(\psi(u,v))_{L^{\infty}}$. *If the positive increasing function* $\psi(u,v)$ *satisfies the condition*

15 $$ (uv)^{-\sigma}\psi(u,v) \textit{ is nondecreasing for some } 0< \sigma< 1, $$

*then the degree of approximation of* $f(x,y)$ *by almost Euler means of its double Fourier series is given by*

$$\begin{aligned} \big\| \tau_{mn}^{rs}(x,y)-f(x,y)\big\| _{p}={}&O \biggl\{ \psi \biggl(\frac{1}{{m+1}},\frac {1}{{n+1}} \biggr)+(n+1)^{-\sigma}\psi \biggl(\frac{1}{m+1},\pi \biggr) \\ &+(m+1)^{-\sigma}\psi \biggl(\pi,\frac{1}{n+1} \biggr)+ \bigl((m+1) (n+1) \bigr)^{-\sigma} \biggr\} . \end{aligned}$$

## Lemmas

We need the following lemmas for the proof of our theorems.

### Lemma 3.1

*Let* $R_{m}^{r}(u)$ *and* $R_{n}^{s}(v)$ *be given by* (10) *and* (11), *respectively*. *Then*

(i) $R_{m}^{r}(u)= O ((1+q_{1})^{m}(m+1) )$ *for* $0< u\leq\frac {1}{m+1}$.
(ii) $R_{n}^{s}(v)= O ((1+q_{2})^{n}(n+1) )$ *for* $0< v\leq \frac{1}{n+1}$.

### Proof

(i) For $0< u\leq\frac{1}{m+1}$, using $\sin(u/2)\geq u/ \pi$ and $\sin mu\leq m\sin u$, we have

16 $$\begin{aligned}[b] \big|R_{m}^{r}(u)\big|={}& \Bigg|\sum_{k=0}^{m} {m\choose k}\frac {q_{1}^{m-k}}{(k+1)}S_{k}^{r}(u) \Bigg| \\ ={}& \Bigg|\sum_{k=0}^{m}{m\choose k} \frac{q_{1}^{m-k}}{(k+1)}\frac{\sin ((k+1)\frac{u}{2})\sin((k+2r+1)\frac{u}{2})}{\sin^{2}(u/2)} \Bigg| \\ \leq{}&\sum_{k=0}^{m}{m\choose k} \frac{q_{1}^{m-k}}{(k+1)}\frac {(k+1)(k+2r+1)\sin(\frac{u}{2})\sin(\frac{u}{2})}{\sin^{2}(u/2)} \\ ={}&\sum_{k=0}^{m}{m\choose k}{q_{1}^{m-k}}(k+2r+1) \\ ={}&(1+q_{1})^{m}(m+2r+1) \\ ={}&O \bigl((1+q_{1})^{m} (m+1) \bigr). \end{aligned} $$

(ii) It can be proved similarly to part (i). □

### Lemma 3.2

*Let* $R_{m}^{r}(u)$ *and* $R_{n}^{s}(v)$ *be given by* (10) *and* (11), *respectively*. *Then*

(i) $R_{m}^{r}(u)= O \Big(\frac{(1+q_{1})^{m}}{(m+1)u^{2}} \Big)$ *for* $\frac{1}{m+1}< u\leq\pi$.
(ii) $R_{n}^{s}(v)= O \Big(\frac{(1+q_{2})^{n}}{(n+1)v^{2}} \Big)$ *for* $\frac{1}{n+1}< v\leq\pi$.

### Proof

(i) For $\frac{1}{m+1}< u\leq\pi$, using $\sin(u/2)\geq u/ \pi$ and $\sin u\leq1$, we have

17 $$\begin{aligned} \big|R_{m}^{r}(u)\big|={}& \Bigg|\sum_{k=0}^{m} {m\choose k}\frac {q_{1}^{m-k}}{(k+1)}S_{k}^{r}(u) \Bigg| \\ ={}& \Bigg|\sum_{k=0}^{m}{m\choose k} \frac{q_{1}^{m-k}}{(k+1)}\frac{\sin ((k+1)\frac{u}{2})\sin((k+2r+1)\frac{u}{2})}{\sin^{2}(u/2)} \Bigg| \\ \leq{}&\sum_{k=0}^{m}{m\choose k} \frac{q_{1}^{m-k}}{(k+1)}\frac{\pi ^{2}}{u^{2}} \\ ={}&\frac{\pi^{2}}{(m+1)u^{2}}\sum_{k=0}^{m} {{m+1}\choose {k+1}}{q_{1}^{m-k}} \\ ={}&\frac{\pi^{2}}{(m+1)u^{2}} \bigl((1+q_{1})^{m+1}-q_{1}^{m+1} \bigr) \\ ={}&O \biggl(\frac{(1+q_{1})^{m}}{(m+1)u^{2}} \biggr). \end{aligned}$$

(ii) It can be proved similarly to part (i). □

## Proof of the main results

### Proof of Theorem 2.1

Using the integral representation of $s_{kl}(x,y)$ given in (2), we have

$$ s_{kl}(x,y)-f(x,y)=\frac{1}{4\pi^{2}} \int_{0}^{\pi} \int_{0}^{\pi}\phi _{x,y}(u,v)D_{k}(u)D_{l}(v) \,du\,dv. $$

Therefore,

$$\begin{aligned} S_{kl}^{rs}(x,y)-f(x,y)={}&\frac{1}{(k+1)(l+1)}\sum _{\gamma=r}^{r+k}\sum_{\mu=s}^{s+l} \bigl(s_{\gamma\mu}(x,y)-f(x,y) \bigr) \\ ={}&\frac{1}{(k+1)(l+1)}\sum_{\gamma=r}^{r+k}\sum _{\mu=s}^{s+l} \int _{0}^{\pi} \int_{0}^{\pi}\frac{\phi_{x,y}(u,v)}{4\pi^{2}}D_{\gamma }(u)D_{\mu}(v) \,du\,dv \\ ={}& \int_{0}^{\pi} \int_{0}^{\pi}\frac{\phi_{x,y}(u,v )}{{4\pi ^{2}}(k+1)(l+1)} \Biggl(\sum _{\gamma=r}^{r+k}D_{\gamma}(u) \Biggr) \Biggl(\sum _{\mu=s}^{s+l}D_{\mu}(v) \Biggr)\,du \,dv. \end{aligned}$$

Now

$$\begin{gathered} \tau_{mn}^{rs}(x,y)-f(x,y) \\ \quad= \Biggl\{ \frac{1}{(1+q_{1})^{m}(1+q_{2})^{n}}\sum_{k=0}^{m} \sum_{l=0}^{n}{m\choose k} {n\choose l}{q_{1}}^{m-k}{q_{2}}^{n-l}S_{kl}^{rs}(x,y) \Biggr\} -f(x,y) \\ \quad=\frac{1}{4\pi^{2}} \int_{0}^{\pi} \int_{0}^{\pi}\frac{\phi _{x,y}(u,v)}{(1+q_{1})^{m}(1+q_{2})^{n}} \Biggl(\sum _{k=0}^{m}\frac{{m\choose k}q_{1}^{m-k}S_{k}^{r}(u)}{(k+1)} \Biggr) \Biggl(\sum _{l=0}^{n}\frac{{n\choose l}q_{2}^{n-l}S_{l}^{s}(v)}{(l+1)} \Biggr)\,du\,dv \\ \quad=\frac{1}{4\pi^{2}} \int_{0}^{\pi} \int_{0}^{\pi}\phi_{x,y}(u,v) \frac {R_{m}^{r}(u)}{(1+q_{1})^{m}}\frac{R_{n}^{s}(v)}{(1+q_{2})^{n}}\,du\,dv, \end{gathered}$$

which, on applying the generalized Minkowski inequality, gives

18 $$\begin{aligned} &\big\| \tau_{mn}^{rs}(x,y)-f(x,y)\big\| _{p} \\ &\quad= \bigg\| \frac{1}{4\pi^{2}} \int_{0}^{\pi}\int_{0}^{\pi}\phi_{x,y}(u,v) \frac {R_{m}^{r}(u)}{(1+q_{1})^{m}}\frac{R_{n}^{s}(v)}{(1+q_{2})^{n}}\,du\,dv \bigg\| _{p} \\ &\quad= \biggl\{ \frac{1}{2\pi} \int_{0}^{2\pi} \int_{0}^{2\pi} \bigg|\frac{1}{4\pi ^{2}} \int_{0}^{\pi} \int_{0}^{\pi}\phi_{x,y}(u,v) \frac {R_{m}^{r}(u)}{(1+q_{1})^{m}}\frac{R_{n}^{s}(v)}{(1+q_{2})^{n}}\,du\,dv \bigg|^{p}\,dx\,dy \biggr\} ^{1/p} \\ &\quad\leq\frac{1}{4\pi^{2}} \int_{0}^{\pi}\int_{0}^{\pi}\big\| \phi_{x,y}(u,v) \big\| _{p}\frac {|R_{m}^{r}(u)|}{(1+q_{1})^{m}}\frac{|R_{n}^{s}(v)|}{(1+q_{2})^{n}}\,du\,dv \\ &\quad=\frac{1}{4\pi^{2}} \biggl\{ \int_{0}^{\frac{1}{(m+1)}} \int_{0}^{\frac {1}{(n+1)}}+ \int_{\frac{1}{(m+1)}}^{\pi}\int_{0}^{\frac{1}{(n+1)}}+ \int _{0}^{\frac{1}{(m+1)}} \int_{\frac{1}{(n+1)}}^{\pi}+ \int_{\frac {1}{(m+1)}}^{\pi}\int_{\frac{1}{(n+1)}}^{\pi}\biggr\} \\ & \qquad{}\times\big\| \phi_{x,y}(u,v)\big\| _{p}\frac{|R_{m}^{r}(u)|}{(1+q_{1})^{m}}\frac {|R_{n}^{s}(v)|}{(1+q_{2})^{n}} \,du\,dv \\ &\quad=\frac{1}{4\pi^{2}}\{I_{1}+I_{2}+I_{3}+I_{4} \},\quad \mbox{say}. \end{aligned}$$

*Proof of part (i)*: Using Lemma 3.1 and (13), we have

19 $$\begin{aligned}[b] I_{1}&= \int_{0}^{\frac{1}{(m+1)}} \int_{0}^{\frac{1}{(n+1)}}\big\| \phi_{x,y}(u,v)\big\| _{p}\frac{|R_{m}^{r}(u)|}{(1+q_{1})^{m}}\frac{|R_{n}^{s}(v)|}{(1+q_{2})^{n}}\,du\, dv \\ &=O \bigl((m+1) (n+1) \bigr) \int_{0}^{\frac{1}{(m+1)}} \int_{0}^{\frac {1}{(n+1)}} \bigl(\omega_{2,x}^{p}(f,u)+ \omega_{2,y}^{p}(f,v) \bigr)\,du\, dv \\ &=O \biggl(\omega_{2,x}^{p} \biggl(f, \frac{1}{m+1} \biggr)+\omega_{2,y}^{p} \biggl(f, \frac{1}{n+1} \biggr) \biggr). \end{aligned} $$

Using Lemma 3.1, Lemma 3.2, (5), and (13), we have

20 $$\begin{aligned}[b] I_{2}&= \int_{\frac{1}{(m+1)}}^{\pi}\int_{0}^{\frac{1}{(n+1)}}\big\| \phi _{x,y}(u,v) \big\| _{p}\frac{|R_{m}^{r}(u)|}{(1+q_{1})^{m}}\frac {|R_{n}^{s}(v)|}{(1+q_{2})^{n}}\,du\,dv \\ &=O \biggl(\frac{n+1}{m+1} \biggr) \int_{\frac{1}{(m+1)}}^{\pi}\int_{0}^{\frac {1}{(n+1)}} \bigl(\omega_{2,x}^{p}(f,u)+ \omega_{2,y}^{p}(f,v) \bigr)\frac{du\, dv}{u^{2}} \\ &=O \biggl(\frac{n+1}{m+1} \biggr) \int_{\frac{1}{(m+1)}}^{\pi}\biggl(\frac {1}{n+1} \biggr) \biggl(\omega_{2,x}^{p}(f,u)+\omega_{2,y}^{p} \biggl(f,\frac {1}{n+1} \biggr) \biggr)\frac{du}{u^{2}} \\ &=O \biggl(\frac{1}{m+1} \biggr) \biggl\{ \int_{\frac{1}{(m+1)}}^{\pi}\frac {\omega_{2,x}^{p}(f,u)}{u^{2}}\,du+ \omega_{2,y}^{p} \biggl(f,\frac{1}{n+1} \biggr) \int_{\frac{1}{(m+1)}}^{\pi}\frac{du}{u^{2}} \biggr\} \\ &=O \biggl(\omega_{2,x}^{p} \biggl(f, \frac{1}{m+1} \biggr)+\omega_{2,y}^{p} \biggl(f, \frac{1}{n+1} \biggr) \biggr). \end{aligned} $$

Similarly, we have

21 $$ I_{3}=O \biggl(\omega_{2,x}^{p} \biggl(f,\frac{1}{m+1} \biggr)+\omega _{2,y}^{p} \biggl(f, \frac{1}{n+1} \biggr) \biggr). $$

Using Lemma 3.2, (5), and (13), we have

22 $$\begin{aligned} I_{4}&= \int_{\frac{1}{(m+1)}}^{\pi}\int_{\frac{1}{(n+1)}}^{\pi}\big\| \phi _{x,y}(u,v) \big\| _{p}\frac{|R_{m}^{r}(u)|}{(1+q_{1})^{m}}\frac {|R_{n}^{s}(v)|}{(1+q_{2})^{n}}\,du\,dv \\ &=O \biggl(\frac{1}{(m+1)(n+1)} \biggr) \int_{\frac{1}{(m+1)}}^{\pi}\int_{\frac {1}{(n+1)}}^{\pi}\bigl(\omega_{2,x}^{p}(f,u)+ \omega_{2,y}^{p}(f,v) \bigr)\frac {du\,dv}{u^{2}v^{2}} \\ &=O \biggl(\frac{1}{(m+1)(n+1)} \biggr) \int_{\frac{1}{(m+1)}}^{\pi}(n+1) \biggl(\omega_{2,x}^{p}(f,u)+ \omega_{2,y}^{p} \biggl(f,\frac{1}{n+1} \biggr) \biggr) \frac{du}{u^{2}} \\ &=O \biggl(\frac{1}{m+1} \biggr) \biggl\{ \int_{\frac{1}{(m+1)}}^{\pi}\frac {\omega_{2,x}^{p}(f,u)}{u^{2}}\,du+ \omega_{2,y}^{p} \biggl(f,\frac{1}{n+1} \biggr) \int_{\frac{1}{(m+1)}}^{\pi}\frac{du}{u^{2}} \biggr\} \\ &=O \biggl(\omega_{2,x}^{p} \biggl(f, \frac{1}{m+1} \biggr)+\omega_{2,y}^{p} \biggl(f, \frac{1}{n+1} \biggr) \biggr). \end{aligned}$$

Collecting (18)–(22), we have

$$ \big\| \tau_{mn}^{rs}(x,y)-f(x,y)\big\| _{p}=O \biggl( \omega_{2,x}^{p} \biggl(f,\frac {1}{m+1} \biggr)+ \omega_{2,y}^{p} \biggl(f,\frac{1}{n+1} \biggr) \biggr), $$

which proves part (i).

*Proof of part (ii)*: Using (19) and (20), we have

23 $$\begin{aligned}& I_{1}=O \biggl(\omega_{2,x}^{p} \biggl(f,\frac{1}{m+1} \biggr)+\omega _{2,y}^{p} \biggl(f, \frac{1}{n+1} \biggr) \biggr), \end{aligned}$$

24 $$\begin{aligned}& I_{2}=O \biggl(\omega_{2,x}^{p} \biggl(f,\frac{1}{m+1} \biggr)+\omega _{2,y}^{p} \biggl(f, \frac{1}{n+1} \biggr) \biggr). \end{aligned}$$

Using Lemma 3.1, Lemma 3.2, (6), and (13), we have

25 $$\begin{aligned}[b] I_{3}&= \int_{0}^{\frac{1}{(m+1)}} \int_{\frac{1}{(n+1)}}^{\pi}\big\| \phi _{x,y}(u,v) \big\| _{p}\frac{|R_{m}^{r}(u)|}{(1+q_{1})^{m}}\frac {|R_{n}^{s}(v)|}{(1+q_{2})^{n}}\,du\,dv \\ &=O \biggl(\frac{m+1}{n+1} \biggr) \int_{0}^{\frac{1}{(m+1)}} \int_{\frac {1}{(n+1)}}^{\pi}\bigl(\omega_{2,x}^{p}(f,u)+ \omega_{2,y}^{p}(f,v) \bigr)\frac {du\,dv}{v^{2}} \\ &=O \biggl(\frac{m+1}{n+1} \biggr) \int_{0}^{\frac{1}{(m+1)}}(n+1) \biggl[\omega _{2,x}^{p}(f,u)+\log\bigl(\pi(n+1)\bigr)\omega_{2,y}^{p} \biggl(f,\frac{1}{n+1} \biggr) \biggr]\,du \\ &=O(m+1) \biggl\{ \int_{0}^{\frac{1}{(m+1)}}{\omega_{2,x}^{p}(f,u)} \,du+\log \bigl(\pi(n+1)\bigr)\omega_{2,y}^{p} \biggl(f, \frac{1}{n+1} \biggr) \int_{0}^{\frac {1}{(m+1)}}\,du \biggr\} \\ &=O \biggl(\omega_{2,x}^{p} \biggl(f, \frac{1}{m+1} \biggr)+\log\bigl(\pi(n+1)\bigr)\omega _{2,y}^{p} \biggl(f,\frac{1}{n+1} \biggr) \biggr). \end{aligned} $$

Using Lemma 3.2, (5), (6), and (13), we have

26 $$\begin{aligned}[b] I_{4}&= \int_{\frac{1}{(m+1)}}^{\pi} \int_{\frac{1}{(n+1)}}^{\pi}\big\| \phi _{x,y}(u,v) \big\| _{p}\frac{|R_{m}^{r}(u)|}{(1+q_{1})^{m}}\frac {|R_{n}^{s}(v)|}{(1+q_{2})^{n}}\,du\,dv \\ &=O \biggl(\frac{1}{(m+1)(n+1)} \biggr) \int_{\frac{1}{(m+1)}}^{\pi}\int_{\frac {1}{(n+1)}}^{\pi}\bigl(\omega_{2,x}^{p}(f,u)+ \omega_{2,y}^{p}(f,v) \bigr)\frac {du\,dv}{u^{2}v^{2}} \\ &=O \biggl(\frac{1}{(m+1)(n+1)} \biggr) \int_{\frac{1}{(m+1)}}^{\pi}(n+1) \biggl[\omega_{2,x}^{p}(f,u)+ \log\bigl(\pi(n+1)\bigr)\omega_{2,y}^{p} \biggl(f, \frac {1}{n+1} \biggr) \biggr]\frac{du}{u^{2}} \\ &=O \biggl(\frac{1}{m+1} \biggr) \biggl\{ \int_{\frac{1}{(m+1)}}^{\pi}\frac {\omega_{2,x}^{p}(f,u)}{u^{2}}\,du+\log\bigl( \pi(n+1)\bigr)\omega_{2,y}^{p} \biggl(f,\frac {1}{n+1} \biggr) \int_{\frac{1}{(m+1)}}^{\pi}\frac{du}{u^{2}} \biggr\} \\ &=O \biggl(\omega_{2,x}^{p} \biggl(f, \frac{1}{m+1} \biggr)+\log\bigl(\pi(n+1)\bigr)\omega _{2,y}^{p} \biggl(f,\frac{1}{n+1} \biggr) \biggr). \end{aligned} $$

Collecting (18), (23)–(26), we have

$$ \big\| \tau_{mn}^{rs}(x,y)-f(x,y)\big\| _{p}=O \biggl( \omega_{2,x}^{p} \biggl(f,\frac {1}{m+1} \biggr)+\log\bigl( \pi(n+1)\bigr)\omega_{2,y}^{p} \biggl(f,\frac{1}{n+1} \biggr) \biggr), $$

which proves part (ii).

In a similar manner, we can prove part (iii) and part (iv). □

### Proof of Theorem 2.2

We have

$$\begin{gathered} \big|\tau_{mn}^{rs}(x,y)-f(x,y)\big| \\ \quad= \bigg|\frac{1}{4\pi^{2}} \int_{0}^{\pi}\int_{0}^{\pi}\phi_{x,y}(u,v) \frac {R_{m}^{r}(u)}{(1+q_{1})^{m}}\frac{R_{n}^{s}(v)}{(1+q_{2})^{n}}\,du\,dv \bigg| \\ \quad\leq\frac{1}{4\pi^{2}} \int_{0}^{\pi}\int_{0}^{\pi}\big|\phi_{x,y}(u,v)\big| \frac {|R_{m}^{r}(u)|}{(1+q_{1})^{m}}\frac{|R_{n}^{s}(v)|}{(1+q_{2})^{n}}\,du\,dv \\ \quad=\frac{1}{4\pi^{2}} \biggl[ \int_{0}^{\frac{1}{(m+1)}} \int_{0}^{\frac {1}{(n+1)}}+ \int_{\frac{1}{(m+1)}}^{\pi}\int_{0}^{\frac{1}{(n+1)}}+ \int _{0}^{\frac{1}{(m+1)}} \int_{\frac{1}{(n+1)}}^{\pi}+ \int_{\frac{1}{(m+1)}}^{\pi}\int_{\frac{1}{(n+1)}}^{\pi}\biggr] \\ \qquad{}\times\big|\phi _{x,y}(u,v)\big| \frac{|R_{m}^{r}(u)|}{(1+q_{1})^{m}}\frac{|R_{n}^{s}(v)|}{(1+q_{2})^{n}}\, du\,dv \\ \quad=\frac{1}{4\pi^{2}}\{I_{1}+I_{2}+I_{3}+I_{4} \},\quad \mbox{say}. \end{gathered}$$

Using (12) and following the proof of Theorem 2.1 with supremum norm, we will get the required result. □

### Proof of Theorem 2.3

Following the proof of Theorem 2.1, using the generalized Minkowski inequality and the fact that $\phi _{x,y}(u,v)\in\operatorname{Lip}(\psi(u,v))_{L^{p}}$ ($p>1$), we have

27 $$\begin{aligned} &\big\| \tau_{mn}^{rs}(x,y)-f(x,y)\big\| _{p} \\ &\quad= \bigg\| \frac{1}{4\pi^{2}} \int_{0}^{\pi}\int_{0}^{\pi}\phi_{x,y}(u,v) \frac {R_{m}^{r}(u)}{(1+q_{1})^{m}}\frac{R_{n}^{s}(v)}{(1+q_{2})^{n}}\,du\,dv \bigg\| _{p} \\ &\quad\leq\frac{1}{4\pi^{2}} \int_{0}^{\pi}\int_{0}^{\pi}\big\| \phi_{x,y}(u,v) \big\| _{p}\frac {|R_{m}^{r}(u)|}{(1+q_{1})^{m}}\frac{|R_{n}^{s}(v)|}{(1+q_{2})^{n}}\,du\,dv \\ &\quad=\frac{1}{4\pi^{2}} \biggl[ \int_{0}^{\frac{1}{(m+1)}} \int_{0}^{\frac {1}{(n+1)}}+ \int_{\frac{1}{(m+1)}}^{\pi}\int_{0}^{\frac{1}{(n+1)}}+ \int _{0}^{\frac{1}{(m+1)}} \int_{\frac{1}{(n+1)}}^{\pi}+ \int_{\frac {1}{(m+1)}}^{\pi}\int_{\frac{1}{(n+1)}}^{\pi}\biggr] \\ &\qquad{}\times M\frac{\psi(u,v)}{(u.v)^{1/p}}\frac{|R_{m}^{r}(u)|}{(1+q_{1})^{m}}\frac {|R_{n}^{s}(v)|}{(1+q_{2})^{n}}\,du\,dv \end{aligned}$$

28 $$\begin{aligned} &\quad=\frac{1}{4\pi^{2}}\{I_{1}+I_{2}+I_{3}+I_{4} \}, \quad\mbox{say}. \end{aligned}$$

Using Lemma 3.1, we have

29 $$\begin{aligned}[b] I_{1}&\leq \int_{0}^{1/{(m+1)}} \int_{0}^{1/{(n+1)}}M\frac{\psi (u,v)}{(u.v)^{1/p}}\frac{|R_{m}^{r}(u)|}{(1+q_{1})^{m}} \frac {|R_{n}^{s}(v)|}{(1+q_{2})^{n}}\,du\,dv \\ &=O \bigl((m+1) (n+1) \bigr)\psi \biggl(\frac{1}{{m+1}},\frac{1}{{n+1}} \biggr) \int_{0}^{1/{(m+1)}} \int_{0}^{1/{(n+1)}}(u.v)^{-1/p}\,du\,dv \\ &=O \biggl\{ \psi \biggl(\frac{1}{{m+1}},\frac{1}{{n+1}} \biggr) \bigl((m+1) (n+1) \bigr)^{1/p} \biggr\} . \end{aligned} $$

Using Lemma 3.1 and Lemma 3.2, we have

30 $$\begin{aligned}[b] I_{2}&\leq \int_{0}^{1/{(m+1)}} \int_{1/{(n+1)}}^{\pi}M\frac{\psi (u,v)}{(u.v)^{1/p}}\frac{|R_{m}^{r}(u)|}{(1+q_{1})^{m}} \frac {|R_{n}^{s}(v)|}{(1+q_{2})^{n}}\,du\,dv \\ &= \int_{0}^{1/{(m+1)}} \int_{1/{(n+1)}}^{\pi}M\frac{(uv)^{-\sigma}\psi (u,v)}{(u.v)^{1/p-\sigma}}\frac{|R_{m}^{r}(u)|}{(1+q_{1})^{m}} \frac {|R_{n}^{s}(v)|}{(1+q_{2})^{n}}\,du\,dv \\ &=O \biggl(\frac{m+1}{n+1} \biggr) \biggl(\frac{\psi(\frac{1}{m+1},\pi)}{ (\frac{1}{m+1}.\pi )^{\sigma}} \biggr) \int_{0}^{1/{(m+1)}} \int _{1/{(n+1)}}^{\pi}\frac{(uv)^{\sigma}}{v^{2}(uv)^{1/p}}\,du\,dv \\ &=O \biggl\{ \psi \biggl(\frac{1}{m+1},\pi \biggr) (m+1)^{1/p}(n+1)^{{1/p}-\sigma} \biggr\} . \end{aligned} $$

Similarly, we have

31 $$ I_{3}=O \biggl\{ \psi \biggl(\pi,\frac{1}{n+1} \biggr) (m+1)^{{1/p}-\sigma }(n+1)^{1/p} \biggr\} . $$

Using Lemma 3.1 and Lemma 3.2, we have

32 $$\begin{aligned}[b] I_{4}&\leq \int_{1/{(m+1)}}^{\pi}\int_{1/{(n+1)}}^{\pi}M\frac{\psi (u,v)}{(u.v)^{1/p}}\frac{|R_{m}^{r}(u)|}{(1+q_{1})^{m}} \frac {|R_{n}^{s}(v)|}{(1+q_{2})^{n}}\,du\,dv \\ &= \int_{1/{(m+1)}}^{\pi}\int_{1/{(n+1)}}^{\pi}M\frac{(uv)^{-\sigma}\psi (u,v)}{(u.v)^{1/p-\sigma}}\frac{|R_{m}^{r}(u)|}{(1+q_{1})^{m}} \frac {|R_{n}^{s}(v)|}{(1+q_{2})^{n}}\,du\,dv \\ &=O \biggl(\frac{1}{(m+1)(n+1)} \biggr) \biggl(\frac{\psi(\pi,\pi)}{(\pi.\pi )^{\sigma}} \biggr) \int_{1/{(m+1)}}^{\pi}\int_{1/{(n+1)}}^{\pi}\frac {(uv)^{\sigma}}{(uv)^{2+1/p}}\,du\,dv \\ &=O \bigl((m+1) (n+1) \bigr)^{1/p-\sigma}. \end{aligned} $$

Collecting (28)–(32), we have

$$\begin{aligned} \big\| \tau_{mn}^{rs}(x,y)-f(x,y)\big\| _{p}={}&O \biggl\{ \bigl((m+1) (n+1) \bigr)^{1/p} \biggl[\psi \biggl(\frac{1}{{m+1}}, \frac{1}{{n+1}} \biggr) \\ &+(n+1)^{-\sigma}\psi \biggl(\frac{1}{m+1},\pi \biggr)+(m+1)^{-\sigma}\psi \biggl(\pi, \frac {1}{n+1} \biggr)\\ &+ \bigl((m+1) (n+1) \bigr)^{-\sigma} \biggr] \biggr\} . \end{aligned}$$

 □

### Proof of Theorem 2.4

We have

33 $$\begin{aligned} &\big|\tau_{mn}^{rs}(x,y)-f(x,y)\big| \\ &\quad= \bigg|\frac{1}{4\pi^{2}} \int_{0}^{\pi}\int_{0}^{\pi}\phi_{x,y}(u,v) \frac {R_{m}^{r}(u)}{(1+q_{1})^{m}}\frac{R_{n}^{s}(v)}{(1+q_{2})^{n}}\,du\,dv \bigg| \\ &\quad\leq\frac{1}{4\pi^{2}} \int_{0}^{\pi}\int_{0}^{\pi}\big|\phi_{x,y}(u,v)\big| \frac {|R_{m}^{r}(u)|}{(1+q_{1})^{m}}\frac{|R_{n}^{s}(v)|}{(1+q_{2})^{n}}\,du\,dv \\ &\quad=\frac{1}{4\pi^{2}} \biggl[ \int_{0}^{\frac{1}{(m+1)}} \int_{0}^{\frac {1}{(n+1)}}+ \int_{\frac{1}{(m+1)}}^{\pi}\int_{0}^{\frac{1}{(n+1)}}+ \int _{0}^{\frac{1}{(m+1)}} \int_{\frac{1}{(n+1)}}^{\pi}+ \int_{\frac{1}{(m+1)}}^{\pi}\int_{\frac{1}{(n+1)}}^{\pi}\biggr] \\ &\qquad{}\times M\psi(u,v)\frac{|R_{m}^{r}(u)|}{(1+q_{1})^{m}}\frac{|R_{n}^{s}(v)|}{(1+q_{2})^{n}}\, du\,dv \\ &\quad=\frac{1}{4\pi^{2}}\{I_{1}+I_{2}+I_{3}+I_{4} \}, \quad\mbox{say}. \end{aligned}$$

Now we can follow the proof of Theorem 2.3 with supremum norm to get the result. □

## Corollaries

If $f\in\operatorname{Zyg}(\alpha,\beta;p)$, then

$$ \omega_{2,x}^{p}(f,u)=O\bigl(u^{\alpha}\bigr) \quad\mbox{and}\quad \omega _{2,y}^{p}(f,v)=O\bigl(v^{\beta} \bigr). $$

For $0<\alpha,\beta<1$,

$$ \int_{\delta}^{\pi}\frac{u^{\alpha}}{u^{2}}\,du=O \bigl( \delta^{\alpha-1} \bigr) \quad\mbox{and}\quad \int_{\delta}^{\pi}\frac{v^{\beta}}{v^{2}}\,dv=O \bigl(\delta ^{\beta-1} \bigr), $$

which implies that $u^{\alpha}$ and $v^{\beta}$ are of the first kind.

For $\alpha=\beta=1$,

$$ \int_{\delta}^{\pi}\frac{u^{\alpha}}{u^{2}}\,du=O \biggl(\log \frac{\pi}{\delta } \biggr) \quad\mbox{and}\quad \int_{\delta}^{\pi}\frac{v^{\beta}}{v^{2}}\,dv=O \biggl(\log \frac{\pi}{\delta} \biggr), $$

which implies that $u^{\alpha}$ and $v^{\beta}$ are of the second kind.

Thus, Theorem 2.1 reduces to the following corollary.

### Corollary 1

*If* $f\in\operatorname{Zyg}(\alpha,\beta;p)$, *then*

$$ \big\| \tau_{mn}^{rs}(x,y)-f(x,y)\big\| _{p}= \left \{ \textstyle\begin{array}{l@{\quad}l} O ((m+1)^{-\alpha}+(n+1)^{-\beta} ), & 0< \alpha, \beta < 1; \\ O ((m+1)^{-\alpha}+\frac{\log(n+1)}{n+1} ), & 0< \alpha< 1, \beta=1; \\ O (\frac{\log(m+1)}{m+1}+(n+1)^{-\beta} ), & \alpha=1, 0< \beta< 1; \\ O (\frac{\log(m+1)}{m+1}+\frac{\log(n+1)}{n+1} ), & \alpha =1,\beta=1. \end{array}\displaystyle \right . $$

For $p=\infty$, the Zygmund class $\operatorname{Zyg}(\alpha,\beta;p)$ reduces to $\operatorname{Zyg}(\alpha,\beta)$. In this case, from Theorem 2.2 we have the following corollary.

### Corollary 2

*If* $f\in\operatorname{Zyg}(\alpha,\beta)$, *then*

$$ \big\| \tau_{mn}^{rs}(x,y)-f(x,y)\big\| _{\infty}= \left \{ \textstyle\begin{array}{l@{\quad}l} O ((m+1)^{-\alpha}+(n+1)^{-\beta} ), & 0< \alpha, \beta < 1; \\ O ((m+1)^{-\alpha}+\frac{\log(n+1)}{n+1} ), & 0< \alpha< 1, \beta=1; \\ O (\frac{\log(m+1)}{m+1}+(n+1)^{-\beta} ), & \alpha=1, 0< \beta< 1; \\ O (\frac{\log(m+1)}{m+1}+\frac{\log(n+1)}{n+1} ), & \alpha =1,\beta=1. \end{array}\displaystyle \right . $$
